# Supplementary material for: Towards reconstructing intelligible speech from the human auditory cortex
Source: Sci Rep. 2019 Jan 29;9:874. doi: 10.1038/s41598-018-37359-z (PMC6351601; doi:10.1038/s41598-018-37359-z)
Supplement: Supplementary file 1 — Supplementary Materials [file 41598_2018_37359_MOESM1_ESM.pdf]

Supplementary Materials for

Towards reconstructing intelligible speech from the human auditory cortex

Hassan Akbari[1,2], Bahar Khalighinejad[1,2], Jose L. Herrero[3,4], Ashesh D. Mehta[3,4], Nima Mesgarani[1,2]

[1]*Mortimer B. Zuckerman Mind Brain Behavior Institute, Columbia University, New York, NY*

[2]*Department of Electrical Engineering, Columbia University, New York, NY*

[3]*Hofstra Northwell School of Medicine, Manhasset, NY, United States*

[4]*The Feinstein Institute for Medical Research, Manhasset, NY, United States*

correspondence to: nima@ee.columbia.edu

This PDF file includes: Tables. T1 to T6, Figures. S1 to S4, audio samples

Input		
**Feature Extraction**		
FCN	CNN	LCN
Flatten()	-	-
Dense (256)<br>BN<br>LeakyReLU(0.25)<br>DropOut(0.3)	Conv2D (32) , [3×3]<br>BN<br>LeakyReLU(0.25)<br>DropOut(0.3)	LCN(1), [5×5]<br>BN<br>LeakyReLU(0.25)<br>DropOut(0.3)
Dense (256)<br>BN<br>LeakyReLU(0.25)<br>DropOut(0.3)	Conv2D (64) , [3×3]<br>BN<br>LeakyReLU(0.25)<br>DropOut(0.3)	LCN(1), [3×3]<br>BN<br>LeakyReLU(0.25)<br>DropOut(0.3)
Dense (256)<br>BN<br>LeakyReLU(0.25)<br>DropOut(0.3)	Conv2D (64) , [3×3]<br>BN<br>LeakyReLU(0.25)<br>DropOut(0.3)	LCN(2), [1×1]<br>BN<br>LeakyReLU(0.25)<br>DropOut(0.3)
-<br>-<br>-<br>-	Conv2D (32) , [1×1]<br>BN<br>LeakyReLU(0.25)<br>DropOut(0.3)	-<br>-<br>-<br>-
-	Flatten()	Flatten()
**Feature Summation**		
Dense (256)<br>BN<br>ELU(alpha=1)		
Output<br>ReLU OR tanh		

Table I. Structure of the proposed FCN, CNN, and LCN neural networks in feature summation and feature extraction networks.

Measure	LR (spectrogram)	DNN (spectrogram)	DNN (Vocoder)	DNN+AEC (Vocoder)
**ESTOI**	0.31	0.40	0.30	**0.47**

Table 2. Objective measures for intelligibility of the reconstructions using linear regression (LR) and DNN with spectrogram and vocoder parameters, with and without the autoencoder (AEC) network. The network with the autoencoder achieves higher reconstruction accuracy.

Bottleneck size	32	64	128	256
**ESTOI**	**0.47**	0.46	**0.47**	**0.47**

Table 3. Objective intelligibility and quality measures of the synthesized speech based on coded-decoded vocoder parameters using AEC with different number of nodes in the bottleneck layer. The number of bottleneck nodes was chosen to maximize the objective and subjective intelligibility, which was highest for both for 256 nodes.

Network	FCN	CNN	LCN	FCN+CNN	FCN+LCN
**ESTOI**	0.45	0.41	0.43	0.42	**0.47**

Table 4. Objective intelligibility measures of the synthesized speech based on coded-decoded vocoder parameters using AEC with different types of network. The feature summation network in all cases is the same (FCN), but different networks architectures were used for feature extraction. Reconstructing the vocoder parameters is best when a FCN+LCN network is used as feature extraction.

Network	FCN	CNN	LCN	FCN+CNN	FCN+LCN
**ESTOI**	**0.40**	0.41	0.37	0.41	0.40

Table 5. Objective intelligibility measures of the synthesized speech based on spectrogram with different types of network. Feature summation network in all cases is the same (FCN). Because no significant difference was observed, we chose the network with lowest complexity which was the FCN model.

<table>
<tr><td colspan="4">Input(513+1+1+1)</td></tr>
<tr><td colspan="4">Dense(512)</td></tr>
<tr><td colspan="4">LeakyReLU</td></tr>
<tr><td colspan="4">Dense(400)</td></tr>
<tr><td colspan="4">LeakyReLU</td></tr>
<tr><td colspan="4">Dense(300)</td></tr>
<tr><td colspan="4">LeakyReLU</td></tr>
<tr><td colspan="4">Dense(256)</td></tr>
<tr><td colspan="4">Tanh (Bottleneck Features)</td></tr>
<tr><td colspan="4">AdditiveGaussianNoise</td></tr>
<tr><td colspan="4">Dense(300)</td></tr>
<tr><td colspan="4">LeakyReLU</td></tr>
<tr><td colspan="4">Dense(400)</td></tr>
<tr><td colspan="4">LeakyReLU</td></tr>
<tr><td colspan="4">Dense(512)</td></tr>
<tr><td colspan="4">LeakyReLU</td></tr>
<tr><td>Dense(512)</td><td>Dense(32)</td><td>Dense(32)</td><td>Dense(32)</td></tr>
<tr><td>LeakyReLU</td><td>LeakyReLU</td><td>LeakyReLU</td><td>LeakyReLU</td></tr>
<tr><td>Dense(513)</td><td>Dense(8)</td><td>Dense(8)</td><td>Dense(8)</td></tr>
<tr><td>ReLU</td><td>LeakyReLU</td><td>LeakyReLU</td><td>LeakyReLU</td></tr>
<tr><td></td><td>Dense(1)</td><td>Dense(1)</td><td>Dense(1)</td></tr>
<tr><td></td><td>ReLU</td><td>ReLU</td><td>ReLU</td></tr>
</table>

Spectrogram Aperiodicity F0 VUV

Table 6. The architecture and the parameters of the autoencoder (AEC) network.

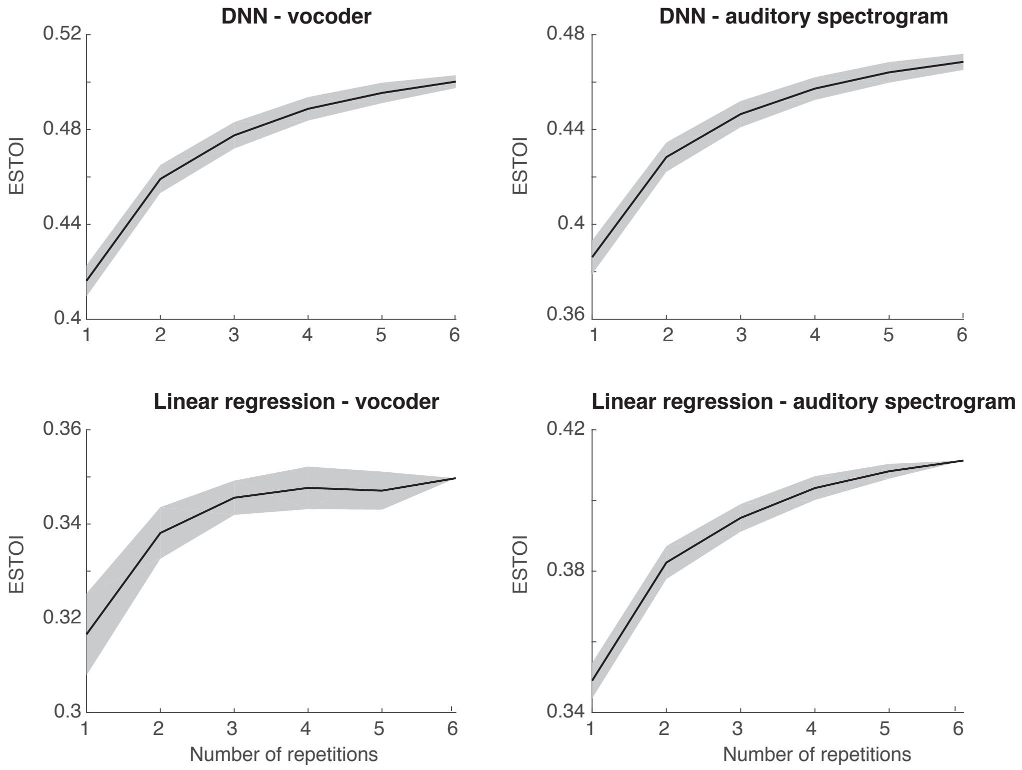

Supplementary Figure 1: ESTOI scores of the four models for speech utterances reconstructed from the averaged neural responses over an increasing number of stimulus repetitions. Shaded lines show the standard deviation.

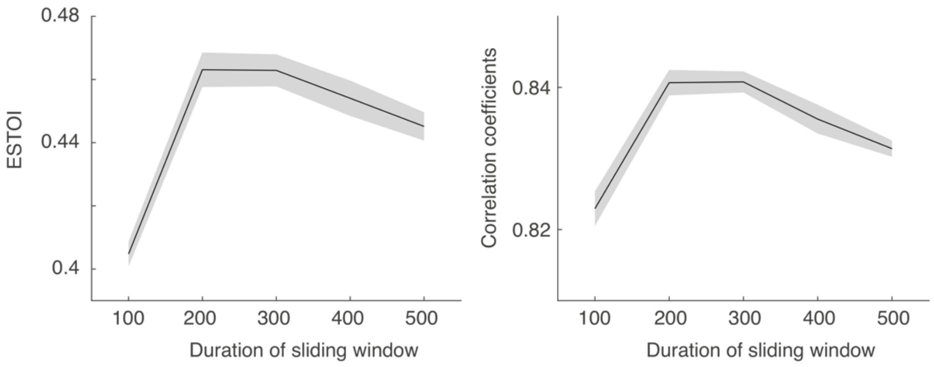

Supplementary Figure 2: Reconstruction accuracy (Pearson's correlation) and ESTOI scores for reconstructed speech sounds when varying the length of the sliding window.

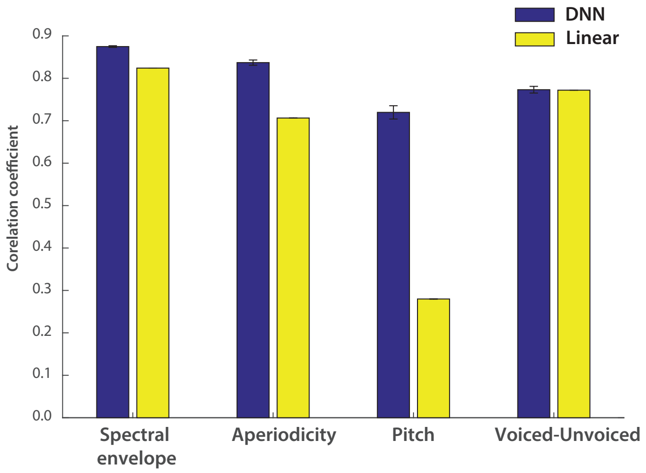

Supplementary Figure 3: Reconstruction accuracy (Pearson's correlation) for individual parameters of the WORLD vocoder model: spectral envelope, aperiodicity, pitch, and voiced-unvoiced.

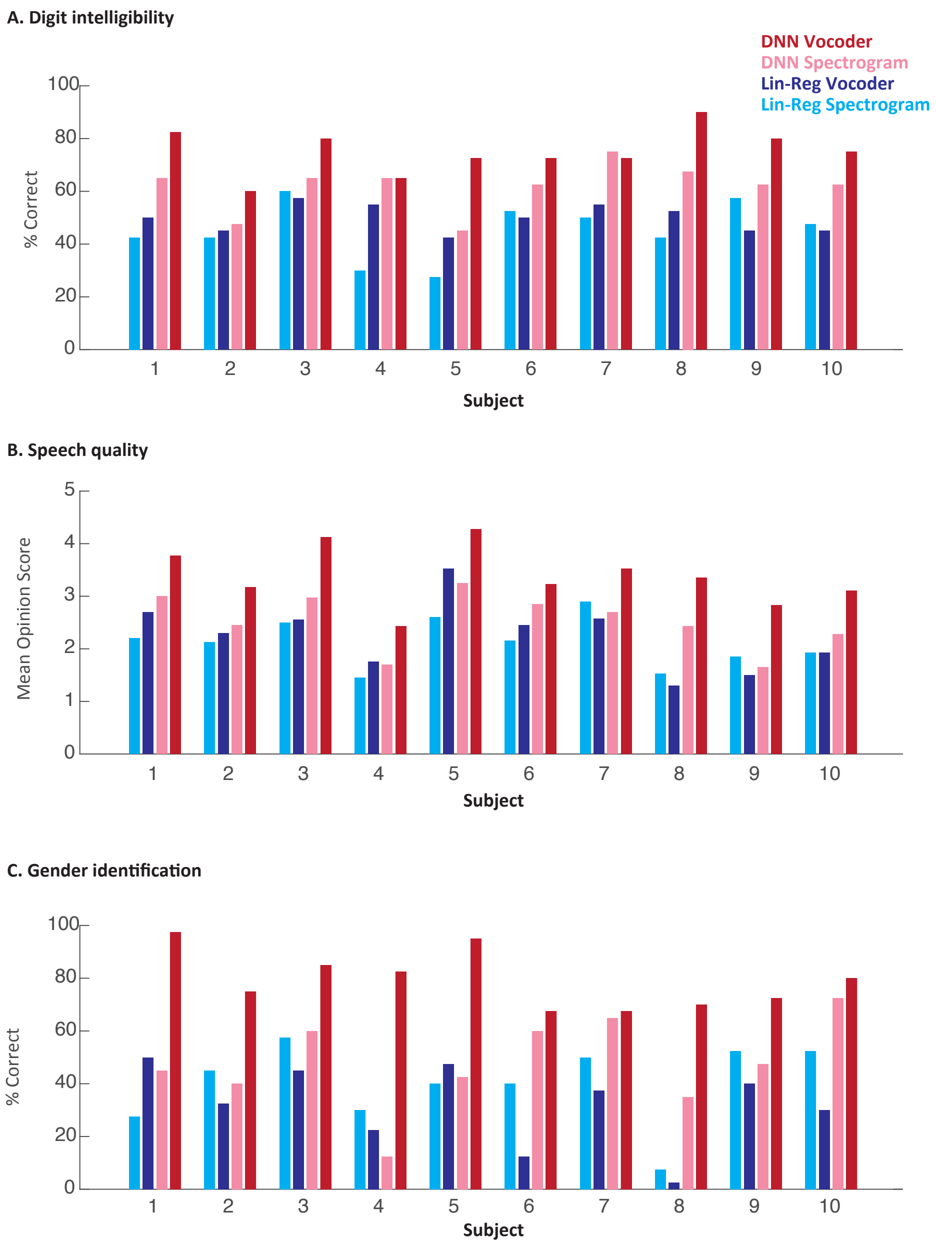

Supplementary Figure 4: Subjective evaluation of the reconstruction models reported separately for the ten listeners. Scores are reported for A) intelligibility, B) quality, and C) gender identification.

Supplemental Movie 1:
The audio files are reconstructed digits from the neural responses in the auditory cortex of listeners. These sounds were used in the subjective intelligibility and quality test (Fig. 3). The digits sounds were taken from a publicly available corpus, TI-46 (See methods). Samples below are from a female (F8) and a male (M5) speaker. For each speaker, the sounds are reconstructed using 4 models: Linear spectrogram, linear vocoder, DNN spectrogram, and DNN vocoder.

F8_ln_aud:	female speaker, linear regression to spectrogram
F8_ln_vocoder:	female speaker, linear regression to vocoder
F8_dnn_aud:	female speaker, DNN to spectrogram
F8_dnn_vocoder:	female speaker, DNN to vocoder

M5_ln_aud:	male speaker, linear regression to spectrogram
M5_ln_vocoder:	male speaker, linear regression to vocoder
M5_dnn_aud:	male speaker, DNN to spectrogram
M5_dnn_vocoder:	male speaker, DNN to vocoder

Supplemental Movie 2:
Example of reconstructing continuous speech from the neural signals which is used for objective evaluations (ESTOI):

Lin_aud_S2: continuous speech, linear regression to spectrogram
Lin_voc_S2: continuous speech, linear regression to vocoder
DNN_aud_S2: continuous speech, DNN to spectrogram
DNN_voc_S2: continuous speech, DNN to vocoder
